# A microbiological and structural analysis of the interplay between sulbactam/durlobactam and imipenem against penicillin-binding proteins (PBPs) of *Acinetobacter* spp.

**DOI:** 10.1128/aac.01627-24

**Published:** 2025-03-04

**Authors:** Balaji Veeraraghavan, Eunjeong Shin, Yamuna Devi Bakthavatchalam, Abi Manesh, Dilip Dubey, Carlo Tascini, Magdalena A. Taracila, Andrea M. Hujer, Michael R. Jacobs, Robert A. Bonomo

**Affiliations:** 1 Department of Clinical Microbiology, Christian Medical College 196380 , Vellore, India; 2 Research Service, Louis Stokes Cleveland Department of Veterans Affairs Medical Center 415208 , Cleveland, Ohio, USA; 3 Department of Medicine, Case Western Reserve University School of Medicine 12304 , Cleveland, Ohio, USA; 4 Department of Infectious Disease, Christian Medical College and Hospital Vellore 30025 , Vellore, India; 5 Institute of Critical Care Medicine, Medanta, Lucknow, India; 6 Department of Infectious Diseases, University of Udine 9316 , Udine, Italy; 7 Department of Pathology, Case Western Reserve University School of Medicine 12304 , Cleveland, Ohio, USA; 8 Clinician Scientist Investigator, Louis Stokes Cleveland Department of Veterans Affairs Medical Center 415208 , Cleveland, Ohio, USA; 9 Departments of Pharmacology, Molecular Biology and Microbiology, Biochemistry, and Proteomics and Bioinformatics, Case Western Reserve University School of Medicine 12304 , Cleveland, Ohio, USA; 10 CWRU-Cleveland VAMC Center for Antimicrobial Resistance and Epidemiology (Case VA CARES) 2546 , Cleveland, Ohio, USA; Johns Hopkins University School of Medicine, Baltimore, Maryland, USA

**Keywords:** sulbactam, durlobactam, beta-lactamase inhibitor, OXA-23, diazabicyclooctane, PBP2, PBP3, PBP1a, sulbactam durlobactam, ATTACK trial

## Abstract

In the ATTACK Phase 3 trial examining the efficacy of sulbactam (SUL)/durlobactam (DUR) to treat primarily *Acinetobacter baumannii* complex (ABC) infections, imipenem (IPM)/cilastatin was added as a common therapy to both the SUL/DUR and the comparator colistin arms. This raised the question of whether the use of IPM in the SUL/DUR arm of the study influenced the efficacy of SUL/DUR. To investigate this issue on a microbiological and molecular level, we performed static concentration time-kill studies and molecular modeling of binding of SUL to PBP1a and PBP3, IPM to PBP1a, PBP2, and PBP3, and DUR to OXA-23 and OXA-51. The time-kill studies performed using carbapenemase- and non-carbapenemase-producing isolates demonstrated synergy between SUL and IPM in the presence of DUR, supporting the notion that the efficacy of the SUL/DUR arm against *Acinetobacter* spp. in the ATTACK trial was enhanced by the addition of IPM. We also hypothesize that the protection of SUL and IPM from OXA carbapenemases by DUR enabled IPM and SUL to synergistically deactivate multiple PBPs (“target redundancy”). Docking simulations supported the favorable binding of SUL to PBP1a and PBP3, resulting in the formation of acyl–enzyme complexes. Molecular docking analysis of OXA carbapenemase enzymes with DUR also revealed favorable interactions. Although clinical trials are warranted, these analyses provide mechanistic support for the addition of IPM to SUL/DUR.

## INTRODUCTION

Durlobactam (DUR) is a diazabicyclooctane (DBO)-class β-lactamase inhibitor (BLI) with a spectrum that includes inhibition of Ambler class A, C, and D enzymes. This spectrum includes the class D OXA carbapenemases found in *Acinetobacter baumannii*–*calcoaceticus* complex (ABC), but does not include Ambler class B metallo-β-lactamases (1). Sulbactam (SUL) is a penicillinate sulfone BLI that alone shows a direct antibacterial activity against ABC due to its penicillin-binding protein (PBP) binding profile, binding preferentially to PBP1a, PBP1b, and PBP3 of ABC (2). However, its activity against the vast majority of the contemporary isolates is compromised due to its vulnerability to ABC-associated class D OXA carbapenemases, certain *Acinetobacter*-derived cephalosporinases (ADC), and TEM-1 β-lactamase (3, 4). Aiming to develop a targeted therapy for infections caused by carbapenemase-producing ABC, DUR was developed as a BLI to inhibit OXA carbapenemases for use in combination with SUL as an *Acinetobacter*-specific combination therapy (5).

A pathogen-specific, multicenter, randomized Phase 3 clinical trial (ATTACK trial) of SUL/DUR formed the basis for its approval by the United States Food and Drug Administration (6). The ATTACK trial was a blinded, non-inferiority study with a 20% non-inferiority margin, wherein the safety and efficacy of SUL/DUR (1/1 g, 3 h infusion, q6h) were compared with that of a standard regimen of colistin. Patients with documented ABC infection [hospital-acquired bacterial pneumonia, ventilator-associated bacterial pneumonia, ventilated pneumonia, or bacteremia] were studied. Since polymicrobial infections are frequent in the patients included in the ATTACK trial, a high-dose regimen of imipenem (IPM)/cilastatin (1/1 g, 1 h infusion, q6h) was added to SUL/DUR to address other important gram-negative pathogens, such as *Pseudomonas aeruginosa* and *Enterobacterales*, as SUL/DUR is active only against ABC (6). Due to the study being a comparative and non-inferiority trial, IPM/cilastatin was also added to the colistin arm, thus serving as a common therapy for both study arms. The primary efficacy endpoint, all-cause mortality (ACM) at day 28 in the microbiological-modified intent to treat population, was 19% in SUL/DUR/IPM compared to 32.3% in colistin/IPM arm (difference: 13.2%, 95% confidence interval: −30%, +3.5%).

Examining these findings more closely, the study design of the ATTACK trial unlocked the possibility of unappreciated synergy on a microbiological and molecular basis between SUL and IPM against ABC owing to their complementary PBP binding profile that could potentially contribute to the efficacy observed in the SUL/DUR/IPM arm. In the triple combination, DUR could protect SUL, as well as IPM, against class D carbapenemases produced by ABC, which is not the case with IPM in the colistin arm. This would enable “protected” IPM to bring about inhibition of its high affinity PBP targets [IC_50_: PBP2 0.08 mg/L (0.26 µM) and PBP1a 0.43 mg/L (1.4 µM)] in an unhindered manner for ABC, capitalizing on its signature feature of PBP2 binding (7). SUL is known to bind to PBP3 (IC_50_: 0.64 mg/L; 2.7 µM) and PBP1b (IC_50_: 0.90 mg/L; 3.8 µM) of ABC (7) and to not bind to PBP1a and PBP2 as well. The combination of SUL with IPM is expected to inhibit multiple essential PBPs (“target redundancy”) of ABC in a complementary manner.

To investigate whether there is a complementary PBP-binding driven, synergistic, pharmacodynamic effect between IPM and SUL in the presence of DUR, we performed MIC and time-kill studies to assess the activity and comparative killing activity of SUL/DUR vs IPM/DUR vs IPM/SUL/DUR, employing five carbapenem-resistant and two carbapenem-susceptible isolates of ABC. The latter strains were included, as any synergistic bactericidal action between SUL and IPM against these isolates could be ascribed to synergistic PBP binding effects, rather than to β-lactamase inhibition. To gain insight into the basis of the multiple PBP inactivation mediated synergy, we performed molecular modeling to assess the possible mechanistic interactions with IPM, SUL, and DUR. The molecular targets involved are penicillin-binding proteins PBP1a, PBP2, and PBP3 and class D β-lactamases OXA-23, OXA-26, and OXA-51.

## RESULTS

### MIC and time-kill studies

The MICs and carbapenemases of the seven study strains are shown in Table 1. All five carbapenem-resistant isolates contained OXA-23 or OXA-26 and OXA-51 with one isolate also containing an NDM carbapenemase. DUR decreased the MICs of IPM and SUL by 4 to >16 fold against the four carbapenem-resistant ABC containing only an OXA carbapenemase, thus demonstrating the protective role of DUR for both IPM and SUL. MICs of IPM/SUL/DUR were similar to those of SUL/DUR. As expected, the two isolates without carbapenemases were susceptible to IPM and SUL.

**TABLE 1:** MICs of study isolates and magnitude of bacterial killing by standalone and combination agents against carbapenem-resistant and susceptible *A. baumannii calcoaceticus* complex

Strains	Carbapenemase	MIC (mg/L)					Magnitude of killing as change in log_10_ CFU/mL at 8 h compared to 0 h (concentration of antibiotics in mg/L)			Difference in magnitude of killing at 8 h between agents shown (log_10_ CFU/mL)	
		IPM	IPM/ DUR^*a*^	SUL	SUL/DUR^*a*^	IPM/ SUL/DUR^*b*^	IPM/DUR^*a*^	SUL/DUR^*a*^	IPM/SUL/DUR^*b*^	IPM/SUL/DUR and IPM/DUR	IPM/SUL/DUR and SUL/DUR
AB 0115	OXA-23, OXA-51	64	16	64	4	4/4	0.17 (2/4)	−0.82 (2/4)	−2.96 (2/2/4)	3.13	2.14
AB 0312	OXA-23, OXA-51	16	1	8	2	0.5/0.5	−1.79 (1/4)	0.66 (1/4)	−2.70 (1/1/4)	0.91	3.36
AB 0399	OXA-23, OXA-51	64	8	32	2	2/2	−0.74 (1/4)	0.22 (1/4)	−2.42 (1/1/4)	1.68	2.64
AB 0459	OXA-23, OXA-51, OXA-58, NDM	128	128	128	32	32/32	1.43 (16/4)	−0.43 (16/4)	−2.27 (16/16/4)	3.7	1.84
AB 0799	OXA-26, OXA-51	>128	8	32	2	2/2	0.53 (1/4)	−1.73 (1/4)	−2.91 (1/1/4)	3.44	1.18
AB 7461	None	0.12	0.06	1	0.5	0.06/0.06	−0.6^*c*^ (0.06)	1.1^*c*^ (0.06)	−2.5^*c*^ (0.06/0.06)	1.9^*d*^	3.6^*e*^
AB 10292	None	0.12	0.12	1	0.5	0.12/0.12	−0.2^*c*^ (0.06)	1.21^*c*^ (0.06)	−3.1^*c*^ (0.06/0.06)	2.9^*d*^	4.3^*e*^

^*a*^ DUR tested at a fixed concentration of 4 mg/L, with results expressed as MICs of IPM or SUL.

^*b*^ DUR tested at a fixed concentration of 4 mg/L with IPM and SUL tested at 1:1 ratio, with results expressed as MICs of IPM/SUL.

^*c*^ Time-kill study performed without DUR.

^*d*^ Difference between IPM/SUL and IPM.

^*e*^ Difference between IPM/SUL and SUL.

**Fig 1:**
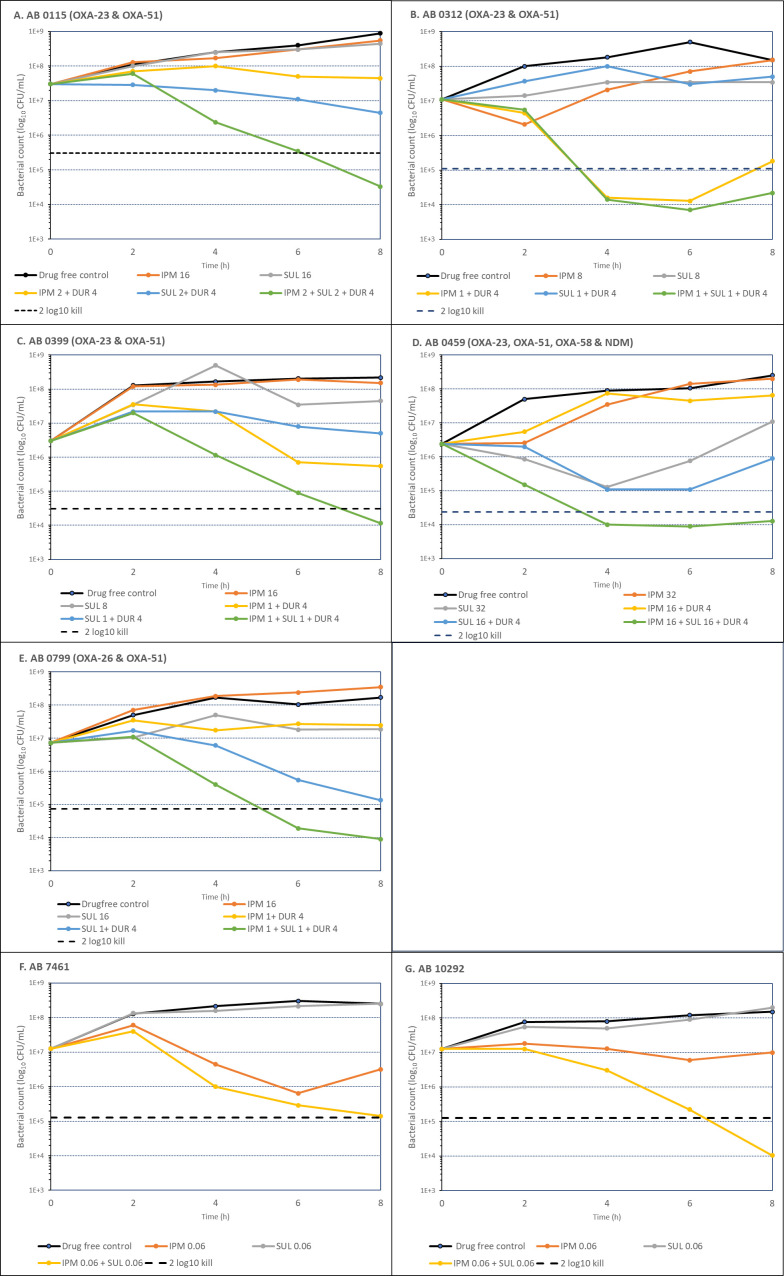
Results of time-kill testing of study isolates against IPM, SUL, and DUR alone and in combination.

Results of time-kill studies are shown in Fig. 1; Table 1. A magnitude of killing of ≥2 log_10_ CFU/mL at 8 h was found only with IPM/SUL/DUR against the carbapenemase-producing isolates and IPM/SUL against the carbapenem susceptible isolates. Additionally, the difference between the magnitude of kill at 8 h against the carbapenemase-producing isolates between IPM/SUL/DUR and IPM/DUR or SUL/DUR ranged from 0.91 to 3.44 log_10_ CFU/mL, with >1 log_10_ CFU/mL for nine of the 10 data sets. The difference between the magnitude of kill at 8 h against the carbapenem susceptible isolates between IPM/SUL and IPM or SUL ranged from 1.9 to 3.6 log_10_ CFU/mL. Additionally, IPM/SUL/DUR demonstrated substantial bactericidal activity at earlier time points (2 to 4 h) in comparison to individual agents, suggesting that this rapid bactericidal action is an outcome of synergistic multiple PBP inhibition, which could be applicable in clinical settings.

### Molecular modeling

**Fig 2:**
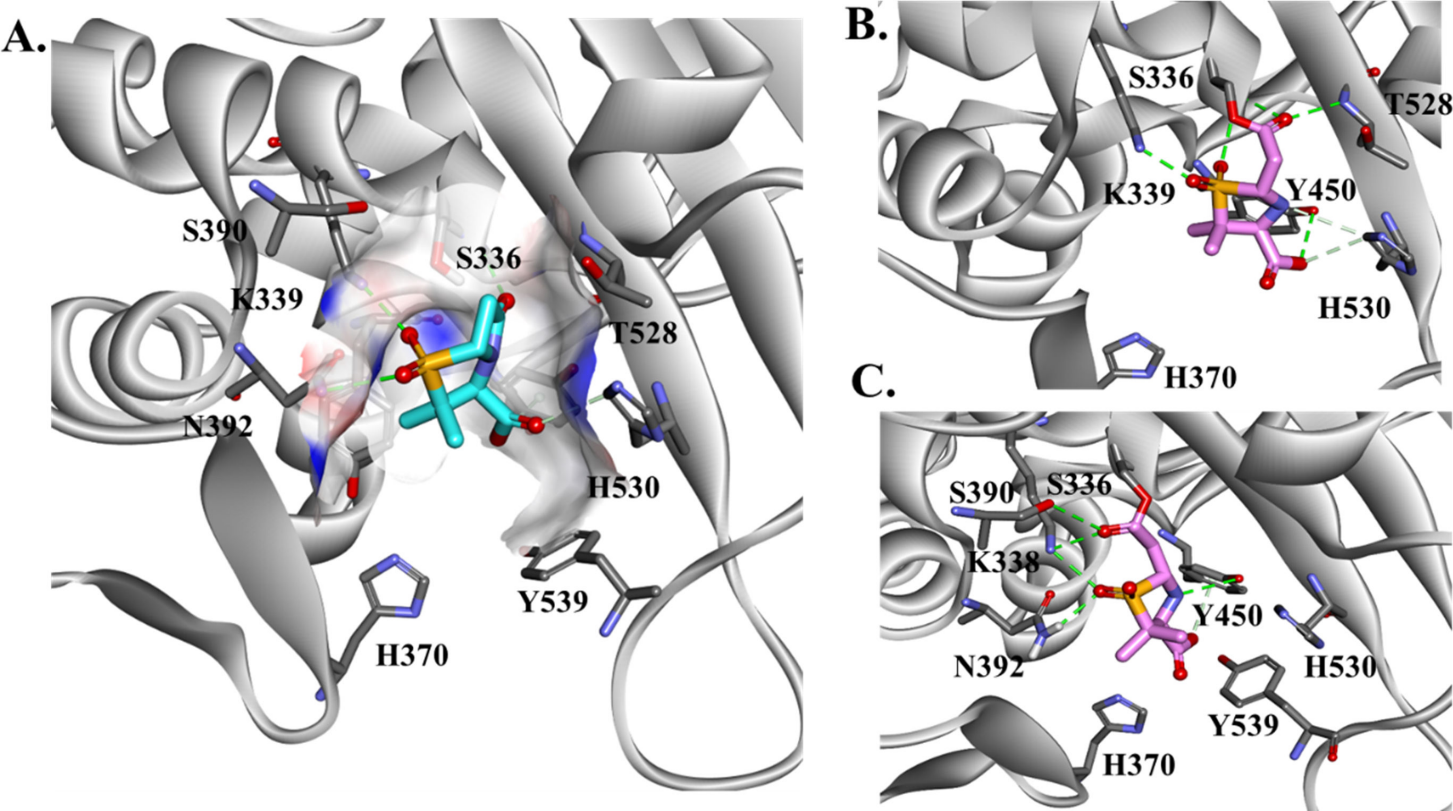
Molecular docking of SUL into the active site of PBP3 as Michaelis–Menten (**A**) and acyl–enzyme complexes (**B and C**). SUL is very well positioned in the active site of PBP3 and ready for acylation. The carbonyl is positioned in the active site, making H-bond interactions with catalytic S336 and S390, K339, and T528. The Y539 position at the entrance of the active site is holding the SUL in a productive conformation. When acyl–enzyme-generated conformations are analyzed, the SUL adopts two different conformations in the active site of PBP3.

**Fig 3:**
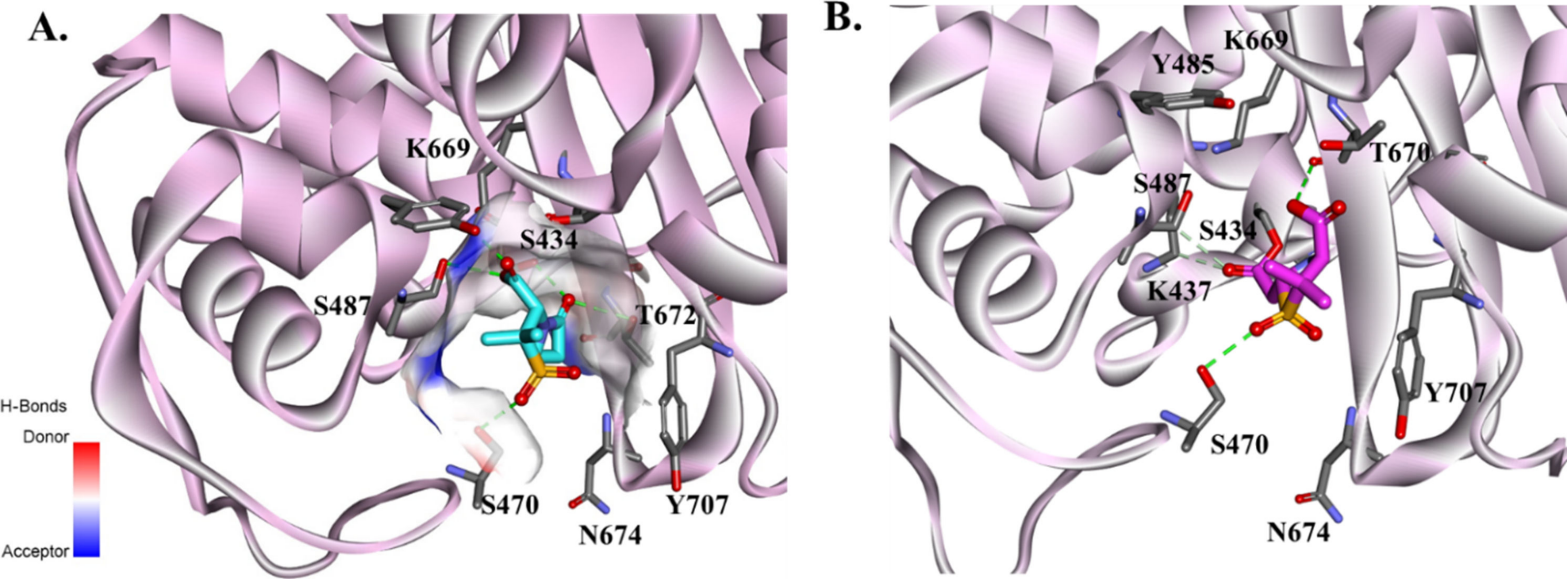
Molecular docking of SUL into the active site of PBP1a as Michaelis–Menten (**A**) and acyl–enzyme complexes (**B**). The SUL carbonyl is positioned toward the catalytic serine S434 and G671, and minimization with constraints was employed to keep the favorable conformation. The carboxyl group is making interaction with K669 and S487. The SO4 group is H-bonded with S470. When the acyl–enzyme is formed, the carbonyl is flipped 180 outside of the oxyanion hole, making interactions with S487 and K437.

**Fig 4:**
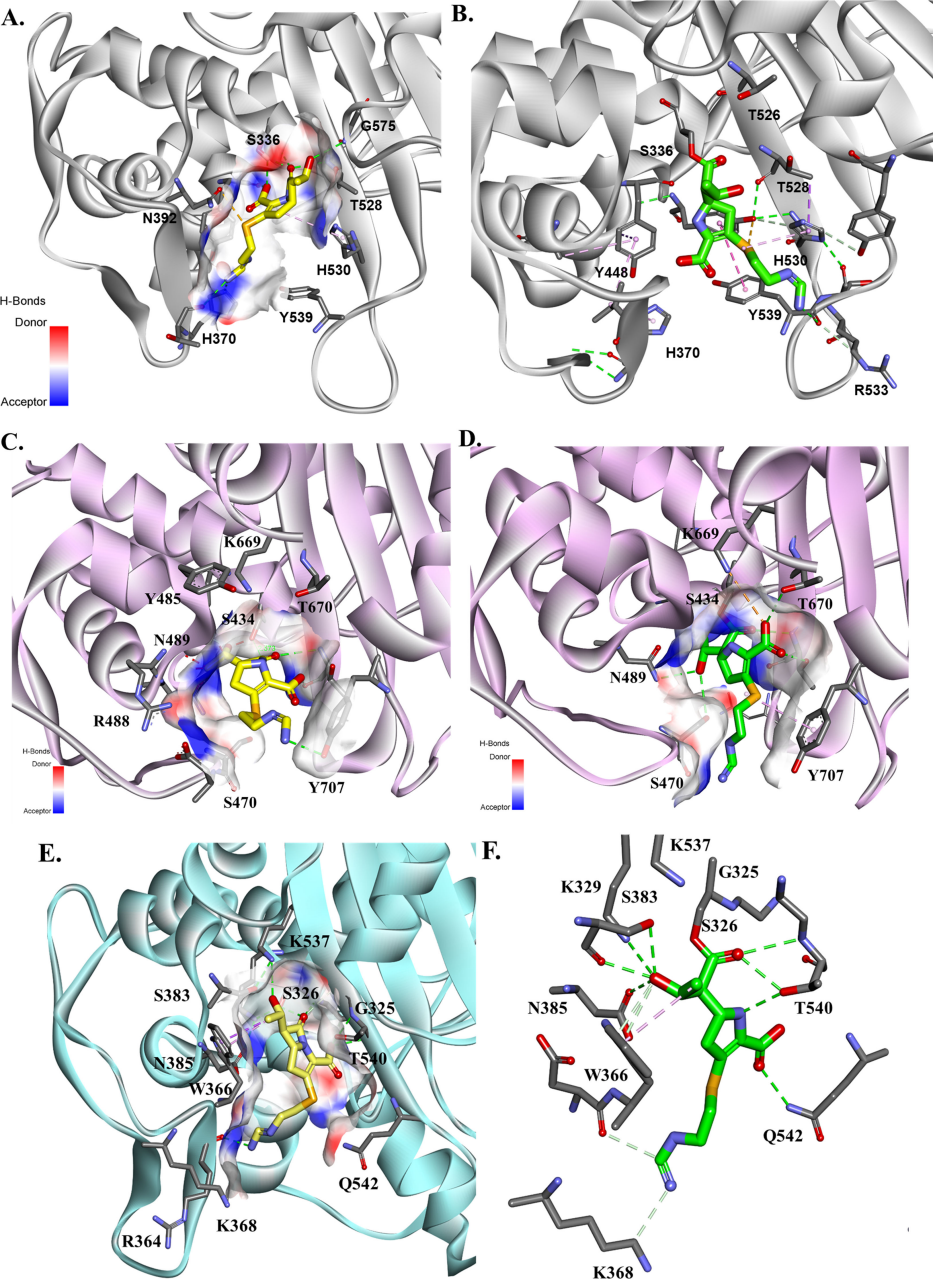
Molecular docking of IPM as Michaelis–Menten (left panels) and acyl–enzymes (right panels) into the active site of PBP3 (**A, B**) PBP1a (**C, D **from PDB #3UDX) and PBP2 (**E, F**). IPM shows different binding patterns in the active sites of the PBPs primarily due to differences in the active site residues.

**Fig 5:**
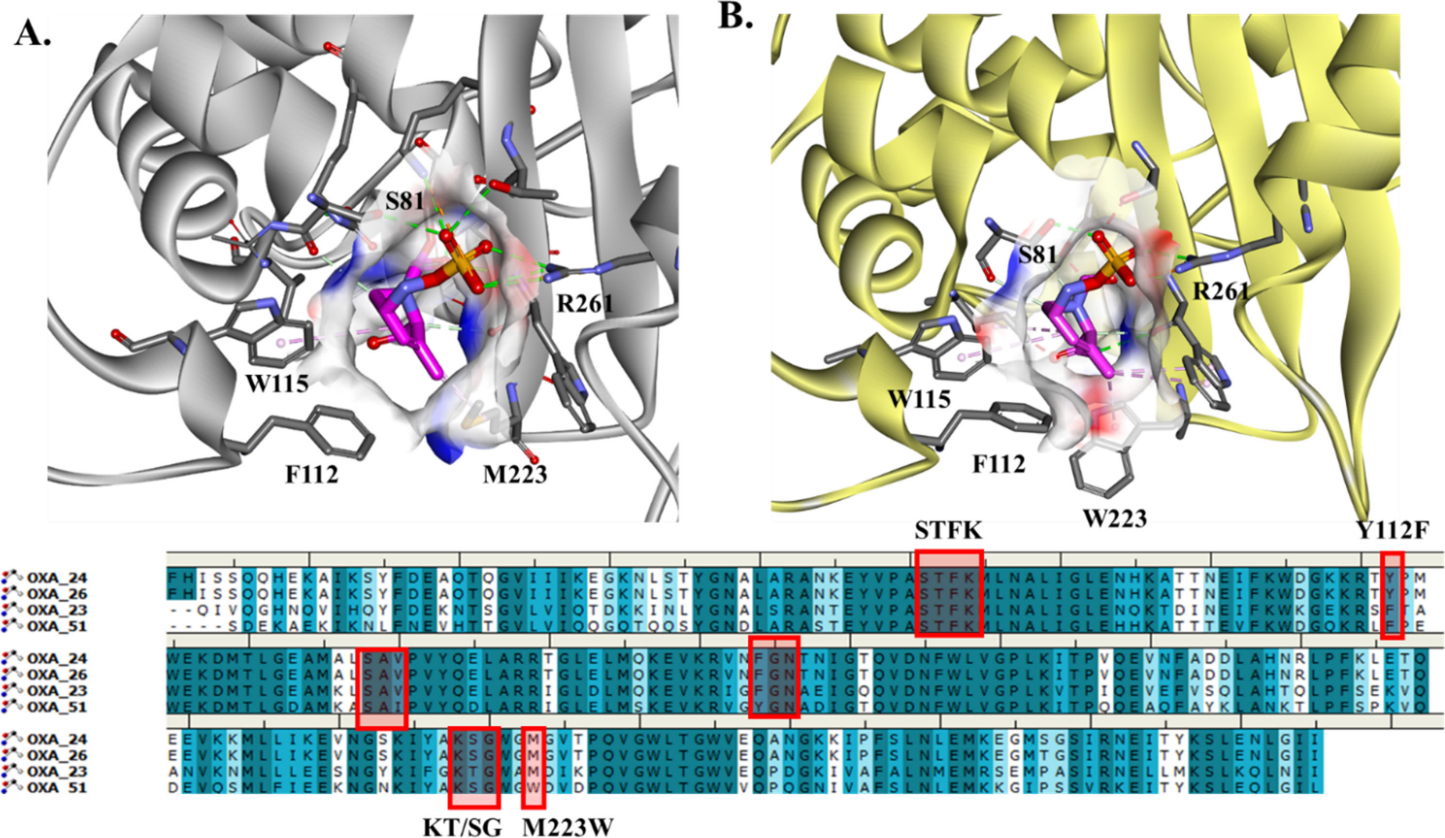
DUR acyl–enzyme complexes with OXA-23 (**A**) and OXA-51 (**B**) based on the crystal structure of OXA-24/-40 (PDB #6MPQ). The bottom panel shows the similarities among binding motifs of the active sites of OXA’s enzymes.

Results of molecular modeling studies are presented in Fig. 2 to 5. The Michaelis–Menten complex of SUL and PBP3 (Fig. 2A) shows that SUL is very well positioned in the active site of PBP3 and ready for acylation. The carbonyl is positioned into the active site making H-bond interactions with the catalytic S336 and S390, K339, and T528. The Y539 position at the entrance of the active site is holding SUL in this productive conformation. The acyl–enzyme-generated conformations show that SUL adopts two different conformations in the active site of PBP3 (Fig. 2B and C). When SUL is docked into the active site of PBP1a (Fig. 3A), the SUL carbonyl is positioned toward the catalytic serine S434 and G671 and making H-bond interactions with K669 and S487. Michaelis–Menten complex minimization with distance constraints is employed to keep the SUL in a favorable conformation. The SO_4_ group is H-bonded with S470. When the acyl–enzyme is formed (Fig. 3B), the carbonyl is flipped 180° outside of the oxyanion hole, resulting in interactions with S487 and K437. This conformation is consistent with the reported low affinity of SUL (>8.5 µM) for PBP1a.

IPM shows different binding patterns in the active site of PBPs (Fig. 4) mostly due to the differences in the active site residues. The GSTXK motif is preserved for all three PBPs. However, PBP1a and PBP2 have an isoleucine (I436), and PBP3 has a methionine (M338) at the X position.

The two histidines (H370 and H530) and Y539 at the entrance of the active site of PBP3 (Fig. 4A and B) may contribute to the lower affinity for IPM as well. The IPM carbonyl is positioned toward the oxyanion hole in both the Michaelis–Menten and acyl–enzyme complexes without making productive interactions (Fig. 4A and B). The positioning of the methyl group flipped toward the catalytic T528 and G575 may impede the H-bond formation of the IPM carbonyl. In PBP1a (Fig. 4C and D), the two histidines are replaced by S470 and N674. The Y707 makes steric interactions with IPM, restraining the potential conformations that IPM can adopt. IPM is positioned very well in the active site of PBP2 (Fig. 4E and F) with a network of H-bonds formed with the PBP2 enzyme. The active site entrance of PBP2 has K368 and N452 replacing the histidines present in the other PBPs. The W366 present in PBP2 makes hydrophobic interactions with the IPM hydroxyethyl group. This positioning of IPM allows for the formation of H-bonds with S383 and K537, and the carboxyl group makes H-bonds with Q542.

Using the crystal structure of OXA-24/-40 and DUR (PDB# 6MPQ), the acyl–enzymes of OXA-23 (Fig. 5A) and OXA-51 (Fig. 5B) were generated. OXA-24/-40 had >98% amino acid identity with OXA-26, with one residue difference (S256T) and approximately 55–60% identity with OXA-23 and OXA-51 (Fig. 5). The typical motif of class D β-lactamase enzymes, STFK, is preserved in OXA-23, OXA-26, and OXA-51. The SXV motif has an isoleucine substitution in OXA-51, and the YGN motif is FGN in OXA-23 and OXA-26. The KTG motif was retained by OXA-23 but replaced by KSG in OXA-26 and OXA-51. Notable differences present in OXA-51 vs OXA-26 are F112 to Y and W223 to M substitutions (Fig. 5).

## DISCUSSION

The objective of our investigation is to investigate the molecular interactions of SUL, IPM, and DUR and assess the impact of this triple combination on MICs . As discussed, IPM was used in both arms of the ATTACK trial, raising the issue of whether IPM may have influenced the outcome of ABC infections in the SUL/DUR arm. A detailed microbiologic analysis of 175 baseline ABC isolates from patients enrolled in ATTACK compared MICs of SUL/DUR and IPM/SUL/DUR (8). SUL/DUR MICs ranged from 0.25/4 to 32/4 mg/L, with MIC_50_ and MIC_90_ values of 2/4 and 4/4 mg/L, respectively; IPM/SUL/DUR MICs ranged from 0.12/0.12/4 to 16/16/4 mg/L, with MIC_50_ and MIC_90_ values of 1/1/4 and 4/4/4 mg/L, respectively. However, critical examination of the correlation between MICs of these agents (shown in Fig. 2B of Ref. [7]) shows a small but consistent lowering of MICs of IPM/SUL/DUR compared to those of SUL/DUR, with seven of eight isolates resistant to SUL/DUR (MICs 16/4 to 32/4 mg/L) being susceptible to IPM/SUL/DUR (MICs 4/4/4 mg/L). These eight SUL/DUR-resistant isolates had mutations, predominantly T526S and A515V, at or near the active site of PBP3, the primary target of SUL, suggesting that the presence of IPM compensates for the compromise in the PBP3 inhibition by SUL. Additional evidence of the synergistic interaction between IPM and SUL was provided in a previous time-kill study of these agents, which showed a synergistic effect at 0.5× and 1× MIC against four isolates of *A. baumannii* after incubation for up to 24 h (9).

Our time-kill studies provide further evidence that the addition of IPM to SUL/DUR is beneficial, with a >2 log_10_ kill at 8 h with IPM/SUL/DUR but not SUL/DUR. IPM/SUL/DUR maintained bacterial killing at 8 h, whereas other treatments, such as monotherapy or SUL/DUR, showed signs of regrowth starting at 8 h, suggesting potential treatment failure. This could be due to the emergence of resistant populations or the presence of slowly replicating bacteria. Notably, the synergistic bactericidal action by the combination of IPM and SUL (in the absence of DUR) occurred, even against carbapenem- and SUL-susceptible strains. In these strains, sub-MIC concentrations of SUL (0.06 mg/L) were ineffective, while 1× MIC of IPM (0.06 mg/L) exhibited a merely bacteriostatic effect. However, the combination of IPM (0.06 mg/L) and SUL (0.06 mg/L) resulted in a significant bactericidal activity, achieving 2.5 and 3.1 log_10_ reductions, respectively, in the bacterial count at 8 h (Fig. 1). This suggests that addition of SUL to IPM enhances the bactericidal action of the combination as a result of complementary PBP binding. This trend is in agreement with earlier reports showing *in vitro* synergy between carbapenems and SUL (9–11). Interestingly, a PBP-based mechanistic synergy between SUL and another PBP2 binding, β-lactamase stable drug, zidebactam, has also been reported against OXA-carbapenemase-producing ABC, even though zidebactam is not an inhibitor of these enzymes (7).

The time-kill studies discussed above demonstrate the advantage of PBP binding-mediated synergy between IPM and SUL against OXA-carbapenemase-producing, as well as SUL/DUR-susceptible, strains. Mechanistically, this effect may be operating *in vivo*; our hypothesis is that the outcomes observed with the SUL/DUR arm of the ATTACK trial against ABC infections might have been assisted by the addition of IPM. This interesting area of exploration was already proposed by Giuliano et al. in their commentary on the ATTACK trial (12, 13). In response, Kaye et al. (6) reported that MIC data that suggested the addition of IPM did not appreciably reduce the MICs of SUL/DUR. Another MIC-based study by McLeod et al. showed only minimal synergy (14). However, it has been demonstrated that the outcome of PBP-based interactions is better revealed by time-kill studies (9, 15), while MIC methods involving observations directly at 18–24 h are not optimal in revealing synergistic bactericidal effects based on multiple PBP binding (16, 17).

The synergistic effect of the combination of IPM with SUL is facilitated by the fast engagement of IPM with PBP2 and PBP1a. The rapid binding of IPM with its targets sets the stage for synergy with SUL, which exhibits complimentary binding to PBP3 and PBP1b (18). The molecular modeling docking analysis further shows the complementary binding of IPM and SUL with different PBPs based on the active site variability and conserved motifs. The GSTXK motif is preserved for all three PBPs analyzed in this work. The methionine (M338) present in PBP3 seems to not affect the IPM or SUL binding (6). The variability in the PBP residues present at the entrance of the active site cavity seems to contribute to the binding affinity of SUL and IPM in a complementary way. We assert that the histidines (H370 and H530) and Y539 present at the entrance of PBP3 facilitate SUL binding and favorably position SUL into the active site of the enzyme (Fig. 2 and 3). When histidines are replaced by serine and asparagine (PBP1) or arginine and glutamine in PBP2, IPM is positioned very well in the active site of PBP enzymes with a network of H-bonds formed. The slightly better binding affinity of IPM for PBP2 versus PBP1a may be due to the presence of Y707 (not present in PBP2 and Y539 in PBP3), which makes steric interactions with IPM, restraining the potential conformations that IPM can adopt (Fig. 4).The complementary effect was demonstrated earlier for IPM, even with isolates producing carbapenem-hydrolyzing β-lactamases (19). The combination with DUR also works synergistically against multiple OXA’s enzyme. The molecular modeling and structural analysis show that the similarities among conserved motifs in the OXA’s enzymes preserved most of the interactions between DUR and OXA enzymes. The methyl group maintains the hydrophobic interactions with positions 223 (W or M) and V221, and the DBO ring is stabilized by W115 and the position 130 residue, which is valine or isoleucine, and the hydrogen sulphate group makes H-bond interactions with R261, S128, and S/T219 and ionic bonds with K218 (Fig. 5). These sites are at the entrance of the active site cavity, forming a “hydrophobic tunnel,” which may play a role in stabilizing the conformation of DUR in the active site of OXA enzymes.

From the pharmacodynamic perspectives, the initial killing phase, even though transient (up to 8 h), does translate into *in vivo* efficacy (20). In this context, in our present study, IPM/SUL/DUR mediated a higher rate and extent of killing against carbapenem-resistant ABC, which is applicable to the clinical scenario. The role of such a mechanism in yielding enhanced bacterial killing was supported by a similar observation for susceptible strains of ABC (no role of DUR as BLI) and against NDM-producing ABC (no role of DUR as BLI), as shown in this study. Therefore, concomitant administration of IPM along with SUL/DUR may be considered in treating serious infections caused by ABC, where these properties are desirable. Based on a few earlier reports of double β-lactam therapy for tackling infections caused by carbapenemase-producing isolates, synergy between IPM and SUL is utilized through a mechanism involving IPM engaging the carbapenemase, thus sparing SUL (9).

Our studies lend support to the Guidance on the Treatment of Antimicrobial-resistant Gram-negative Infections (21) recommendation that the preferred treatment of carbapenem-resistant ABC infections is SUL–DUR in combination with a carbapenem (imipenem–cilastatin or meropenem) (21). The rationale for this recommendation is that the potential benefit may be related to the additional PBPs that are targeted with multiple β-lactams, with SUL preferentially binding to PBP1 and PBP3, while IPM preferentially binds to PBP2.

In conclusion, our study provides additional evidence that the addition of IPM to SUL/DUR is beneficial due to DUR protecting IPM and SUL from the activity of OXA-carbapenemases, thereby facilitating complementary inactivation of multiple PBPs. Furthermore, molecular modeling aided in elucidating the binding mechanisms of the essential PBPs for IPM and SUL, leading to the formation of acyl complexes. The potential also exists to apply this unique strategy to other difficult to treat infections, using zidebactam, which has affinity for PBP2 of *Enterobacterales*, *P. aeruginosa*, and *A. baumannii*, and nacubactam, which has affinity for PBP2 of *Enterobacterales*.

## MATERIALS AND METHODS

### MIC determination and static concentration time-kill studies

Seven ABC isolates were selected for testing, namely, five carbapenem-resistant (AB 0115, AB 0312, AB 0399, AB 0459, and AB 0799) and two carbapenem-susceptible ABC (AB 7461 and AB 10292) (Table 1). IPM was purchased as its commercial formulation, while SUL was procured from Medkoo Biosciences, Durham, North Carolina and DUR from TargetMol Chemicals Inc., Boston, MA. All agents were diluted in sterile distilled water.

MICs of IPM, SUL, IPM/DUR, SUL/DUR, and IPM/SUL/DUR were determined following the Clinical Laboratory and Standards Institute Guidelines (22). DUR was combined with IPM or SUL at 4 mg/L. IPM/SUL/DUR was tested at a 1:1 ratio of IPM:SUL with a fixed concentration of DUR of 4 mg/L. MICs were interpreted as follows (23): 1) IPM, ≤2 mg/L, susceptible, 4 mg/L, intermediate, ≥8 mg/L, resistant; and 2) SUL/DUR, ≤4/4 mg/L, susceptible, 8/4 mg/L, intermediate, ≥16/4 mg/L, resistant.

Time-kill studies were conducted over an 8-h period in tubes containing Mueller–Hinton II broth (BD Difco, UK) to evaluate the efficacy of IPM, SUL, and DUR both individually and in combination against the seven study strains using the same concentrations and ratios of agents used for MIC determination. Tubes were incubated with shaking (120 rpm) at 37°C. Initial inocula used in time-kill studies ranged from 6.38 to 7.48 log_10_ CFU/mL. Viable counts were assessed immediately prior to dosing (0 h), and then at 2 h intervals for a total duration of 8 h. To prevent antibiotic carry-over, all samples were serially diluted 10-fold for viable count determination. The viable count procedure involved plating of 10 µL of undiluted or diluted samples onto tryptic soy agar plates (BD Difco, UK), which were incubated overnight at 37°C. Dilutions with 30–300 colonies per plate were used to determine bacterial counts, with a lower limit of detection of 3 × 10^2^ CFU/mL. For monotherapy of IPM or SUL for carbapenem-resistant ABC, we used 8–16 mg/L, as this range falls within the clinically achievable concentration. For combination therapy of carbapenem-resistant ABC, IPM and SUL were tested at lower concentrations of 1–2 mg/L to evaluate potential synergistic effects. SUL concentrations used in time-kill studies were at 0.5× MIC of the SUL component of SUL/DUR for carbapenem-resistant ABC. A positive combination effect (synergy) was considered when there was at least one log_10_ kill by IPM/SUL/DUR compared to that of either IPM/DUR or SUL/DUR.

### Molecular modeling and docking

The *A. baumannii* PBP2 and PBP3 homology models were created using the crystal structure template of PBP2 (PDB #7ZG8) (24) and PBP3 (PDB #3UE3) (25). The missing residues (V223–K238/K544–A556 of PBP2 and V97–G185/I237 L245 of PBP3) were reconstructed using SWISS-MODEL homology-modeling server accessible via the ExPASy web server (26). For molecular modeling studies of the PBP1a enzyme, the apo enzyme crystal structure (PDB #3UDF) and PBP1a co-crystalized with IPM structure (PDB #3UDX) were used. The missing residues from the PBP1a crystal structure were reconstructed similarly as for the PBP3 enzyme using SWISS-MODEL homology-modeling server. The structures were further minimized using Discovery Studio software (BIOVIA DS Client 2020, San Diego, CA). Conjugate gradient method with an RMS gradient of 0.001 kcal/(mol × Å) was employed. Generalized born with a simple switching solvation model was used, and long-range electrostatics were treated using a Particle Mesh Ewald method with periodic boundary condition. The SHAKE algorithm was applied. The crystal structures of OXA-23 (PDB #4K0X) (27) and OXA-51 (PDB #4ZDX) (28) were used to create the complexes with DUR. The acylated DUR from OXA-24/-40-DUR complex (PDB #6MPQ) (29) was manually docked into the active site of the OXA enzymes.

The intact and acyl SUL and IPM were built and docked into the active site of PBP1a, PBP2, and PBP3 enzymes. The CDOCKER protocol was used to dock the compounds into the active site of PBP’s enzymes. The protocol uses a CHARMm-based molecular dynamics scheme to dock ligands into a receptor-binding site. The generated poses were analyzed, and the best ranked were used to create the Michaelis–Menten and acyl–enzyme complexes and further minimized.
